# Genetic Diversity of Fowlpox Virus and Putative Genes Involved in Its Pathogenicity

**DOI:** 10.1128/spectrum.01415-22

**Published:** 2022-09-08

**Authors:** Hye-Ryoung Kim, Il Jang, Hye-Soon Song, Si-Hyeon Kim, Hyeon-Su Kim, Yong-Kuk Kwon

**Affiliations:** a Avian Disease Division, Animal and Plant Quarantine Agency, Gimcheon-si, Gyeongsangbuk-do, Republic of Korea; University of Georgia

**Keywords:** fowlpox virus, comparative analysis, pathogenicity, reticuloendotheliosis virus, comparative genomics

## Abstract

To determine the genomic variations of fowlpox virus (FPV)—the largest, very ancient, and still harmful avian virus—the complete genomes of 21 FPVs were analyzed. The genomes showed low genetic diversity relative to their overall size. Our studies revealed that FPVs could phylogenetically be divided into two clades, based on their regional distribution, and comparative analysis showed that 40 putative proteins of FPV were associated with geographic differences in viruses, viral pathogenicity, or the onset of diphtheritic lesions. The strain, classified into a subgroup different from others in the genomic analysis, showed relatively low pathogenicity in chickens, and the onset of diphtheritic lesions was observed to be caused only by the specific strain. Despite genetic differences, some commercial vaccines are protective against virulent strains, and intact reticuloendotheliosis virus inserted into field FPV strains was activated but there was no enhancement of the pathogenicity of FPV. These findings will expand our knowledge of the viral proteome and help us understand the pathogenicity of FPV.

**IMPORTANCE** This study aims at determining molecular candidates using comparative genomics to differentiate between the diphtheritic and cutaneous forms of FPV infection, in addition to their association with the pathogenicity of the virus. Full-genomic analyses of multiple fowlpox strains, including field viruses, isolated between 1960s and 2019, and vaccine strains showed the genetic diversity due to regional differences. Comparative genomic analysis offered the clues related to viral virulence. We believe that our study makes a significant contribution to the literature because we are the first to perform such an elaborate study that compares 21 FPVs to study and highlight their diversity, despite the high level of homology between them. Our results shall help provide insights for tackling FPV that has been taking a toll on the poultry for years now.

## INTRODUCTION

Fowlpox (FP) is a viral disease of poultry. It was first discovered by Bollinger in 1873 and its vaccine was introduced in 1918. Fowlpox virus (FPV), the causative agent of FP, belongs to the genus Avipoxvirus of the *Chordopoxvirinae* subfamily of the *Poxviridae* family and infects chickens and turkeys. Smallpox, a disease in humans, was eradicated in 1980, but FP continues to significantly affect gallinaceous birds and is prevalent worldwide, regardless of climate, age, and location (backyard or commercial farming flocks). Attenuated vaccines have been used to control FP, but the recurrence in previously vaccinated flocks is a matter of concern (1, 2).

FPV vaccines consist of live attenuated strains of FPV or of other avian poxviruses like the pigeonpox virus (1). The origins and development of FPV commercial vaccines by numerous veterinary pharmaceutical companies are enigmatic since most of them were licensed decades ago before molecular methods for characterization became established. To date, there is no information on the whole-genome sequences of these vaccine strains or their original strain.

There are various unresolved questions about FP, despite many studies over approximately a hundred and 50 years. First, several questions about the genetic characteristics of FPV remain unanswered because of their large genome size (~300 kbp, kbp). After the first genome was sequenced in the year 2000, the complete genomes of a few FPV strains were sequenced and all known FPV were classified by the phylogeny of the 4b gene (3–6). Dozens of hypothetical proteins and the genetic characteristics responsible for these pathogenic differences have been identified, but their functions remain to be elucidated. Second, most FPVs isolated from the field have an insertion of an almost complete genome of the reticuloendotheliosis virus (REV); this insertion is thought to enhance its pathogenicity and was responsible for FP outbreaks by REV-induced immunosuppression, even in vaccinated flocks (1, 7). It is still unclear how REV, as a provirus in the FPV genome, has a role in influencing the pathogenicity of FPV. Third, there is little information about the difference between the cutaneous and diphtheritic forms. The cutaneous form of the disease is mild and common, characterized by hyperplasia of the epidermis, but the diphtheritic form, characterized by fibronecrotic and proliferative lesions on the mucosal membrane of the larynx, trachea, and esophagus, often causes up to 15% mortality in chickens by asphyxiation (8). Until now, it was unclear whether diphtheritic pox was caused by infection via different routes or by different strains.

This study was performed to investigate the sequence diversity of 21 FPV strains and employed comparative genomics for subsequently investigating whether the difference in pathogenicity was due to genetic variation or the integration of other viruses. To determine whether pathogenicity differs depending on virus strains or routes of infection and to ensure the efficacy of vaccines to protect against virulent viruses, the ability of three field strains and three commercial vaccines was evaluated.

## RESULTS

### The fowlpox virus genome.

**FIG 1:**
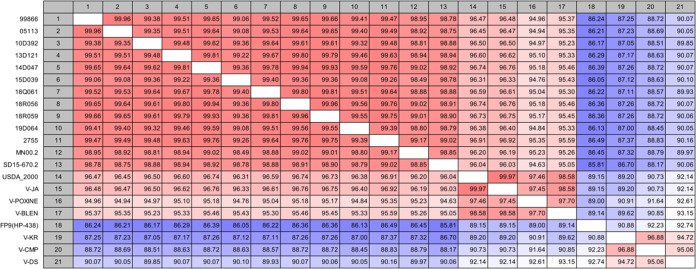
Percent identity matrix of the complete nucleotide sequences of 21 FPVs.

**FIG 2:**
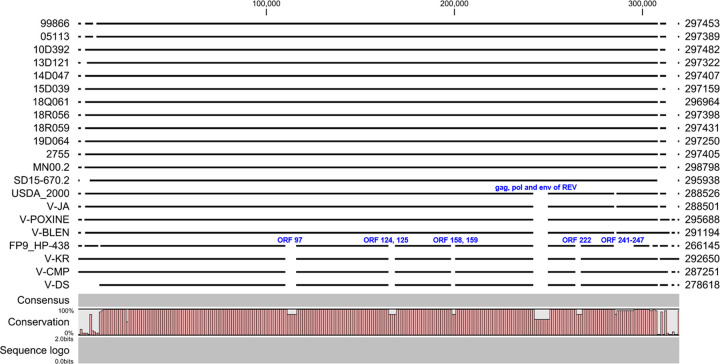
Alignment of complete genomes of 21 FPVs.

Complete genomes of 17 FPV strains – of 11 viruses isolated in the Republic of Korea from 1999 to 2019, strain 2755 obtained from Michigan University in the United States in the 1960s (9) and six vaccine strains available in the market were sequenced while four reported FPV genome sequences – the USDA_2000, MN00.2, SD15-670.2, and FP9 – were used (Table 1). Eleven viruses isolated from clinical specimens and six vaccine strains, all contained linear genomic DNA of approximately 287–297 kbp with 5′- and 3′-ends adjoining inverted terminal repeat (ITR) regions. Percent identities were in the range of 89.15 and 99.97% in the pairwise comparison between 20 strains and USDA_2000 (NCBI accession number: AF198100); (3). Prokka annotation (10) indicated that the coding region sequences of the 21 FPV genome sequences added four publicly available sequence isolates containing 253 to 265 open reading frames (ORFs) among the strains. The USDA_2000 is known to have 260 ORFs and 37 hypothetical proteins of unknown function that were annotated using the Glimmer tool based on local BLAST (3); however, Prokka analyses revealed 261 ORFs and 17 hypothetical proteins based on a nonredundant protein sequence database (nr DB). The intact sequence of REV, including three ORFs (*gag*, *pol*, and *env* genes), was inserted in 14 strains that excluded six vaccine strains and USDA_2000. A total of 10 strains isolated from the Republic of Korea from 1999 to 2019 showed more than 99.08% identity despite the difference in FP onset time, and the genome identities between Korean strains and U.S. field strains were more than 98.75% despite their geographic differences. However, three vaccine strains (V-DS, V-CMP, and V-KR) and FP9 showed relatively low identity of less than 90% with field strains (Fig. 1). Alignment of complete genomes displayed gene-loss areas involving ORFs 97, 124, 125 158, 159, 222, the end regions of FPV genome, and *gag*, *pol*, and *env* genes of the inserted REV provirus (Fig. 2). The pan-genome analysis pipeline (PGAP) analysis showed that the genome of the 21 FPV strains comprised 297 gene clusters, of which 228 clusters were common in all strains (called core genes), and the others comprised the truncated, duplicate, or accessory genes. Although it had been reported that the FPV strains have very high similarity among themselves, with an increase in the number of viruses compared, the number of core genes decreased and the number of gene clusters increased, showing an “open” pangenome pattern.

**TABLE 1:** Prokka annotation features of 21 FPVs analyzed^*a*^

No.	Fowlpox strain	Origin^*b*^	Yr. of isolation	Phenotype	Sequences determined (bp)	Percent identity^*c*^ (%)	Putative ORFs	Insertion of intact REV	GenBank accession no.^*d*^
1	99866	ROK, Ck	1999	Diphtheritic	297,453	96.47	263	Yes	MW558075
2	05113	ROK, Ck	2005	Cutaneous	297,389	96.45	263	Yes	MW558074
3	10D392	ROK, Ck	2010	Cutaneous	297,482	96.50	256	Yes	MW558065
4	13D121	ROK, Ck	2013	Cutaneous	297,322	96.60	264	Yes	MW558066
5	14D047	ROK, Ck	2014	Cutaneous	297,407	96.74	262	Yes	MW558067
6	15D039	ROK, Ck	2015	Cutaneous	297,159	96.31	257	Yes	MW558068
7	18Q061	ROK, Ck	2018	Diphtheritic	296,964	96.59	262	Yes	MW558069
8	18R056	ROK, Ck	2018	Diphtheritic	297,398	96.74	263	Yes	MW558070
9	18R059	ROK, Ck	2018	Diphtheritic	297,431	96.73	263	Yes	MW558071
10	19D064	ROK, Ck	2019	Cutaneous	297,250	96.38	262	Yes	MW558072
11	2755	USA, Ck	<1963	unk	297,405	96.91	265	Yes	MW558073
12	USDA_2000^*e*^	USA, Ck	1999	unk	288,872		261	No	AF198100
13	MN00.2^*e*^	USA, Tk	2000	unk	298,798	96.20	264	Yes	MH709124
14	SD15-670.2^*e*^	USA, Tk	2015	Cutaneous	295,937	96.04	265	Yes	MH734528
15	FP9^*e*^	Germany Munich	1960s	unk	266,145	89.15	253	No	AJ581527
**No.**	**Vaccine strain**	**Origin**	**Yr. of permit**	**Manufacturer**	**Sequences determined (bp)**	**Percent identity (%)**	**Putative ORFs**	**Insertion of intact REV**	**GenBank accession no.**
16	V-CMP	2755	1976	Komipharm	287,251	90.73	259	No	MW558078
17	V-DS	2755	1969	DSMbio	287,618	92.14	258	No	MW558079
18	V-KR	2755	1985	KBNP	292,650	89.20	260	No	MW558080
19	V-JA	Cutter	1974	CAVAC	288,501	99.97	264	No	MW558081
20	V-POXINE	unk	1982	Zoetis	295,688	97.46	262	No	MW558076
21	V-BLEN	Cutter	1980	Boehringer-Ingelheim	291,194	98.58	256	No	MW558077

a Virus identified, origin, sequence size, GenBank accession number.

b ROK, Republic of Korea; Ck, chicken; Tk, turkey; unk, unknown.

c Percent identities were calculated by the pairwise comparison between 20 strains and USDA_2000.

d Complete genome sequences of 17 FPVs have been submitted to GenBank under accession numbers MW558065–MW558081.

e We used reported nucleotide sequences for fowlpox USDA_2000, FWPV-MN00.2, FWPV-SD15-670.2, and FP9(HP-438p).

### Phylogenetic inference.

**FIG 3:**
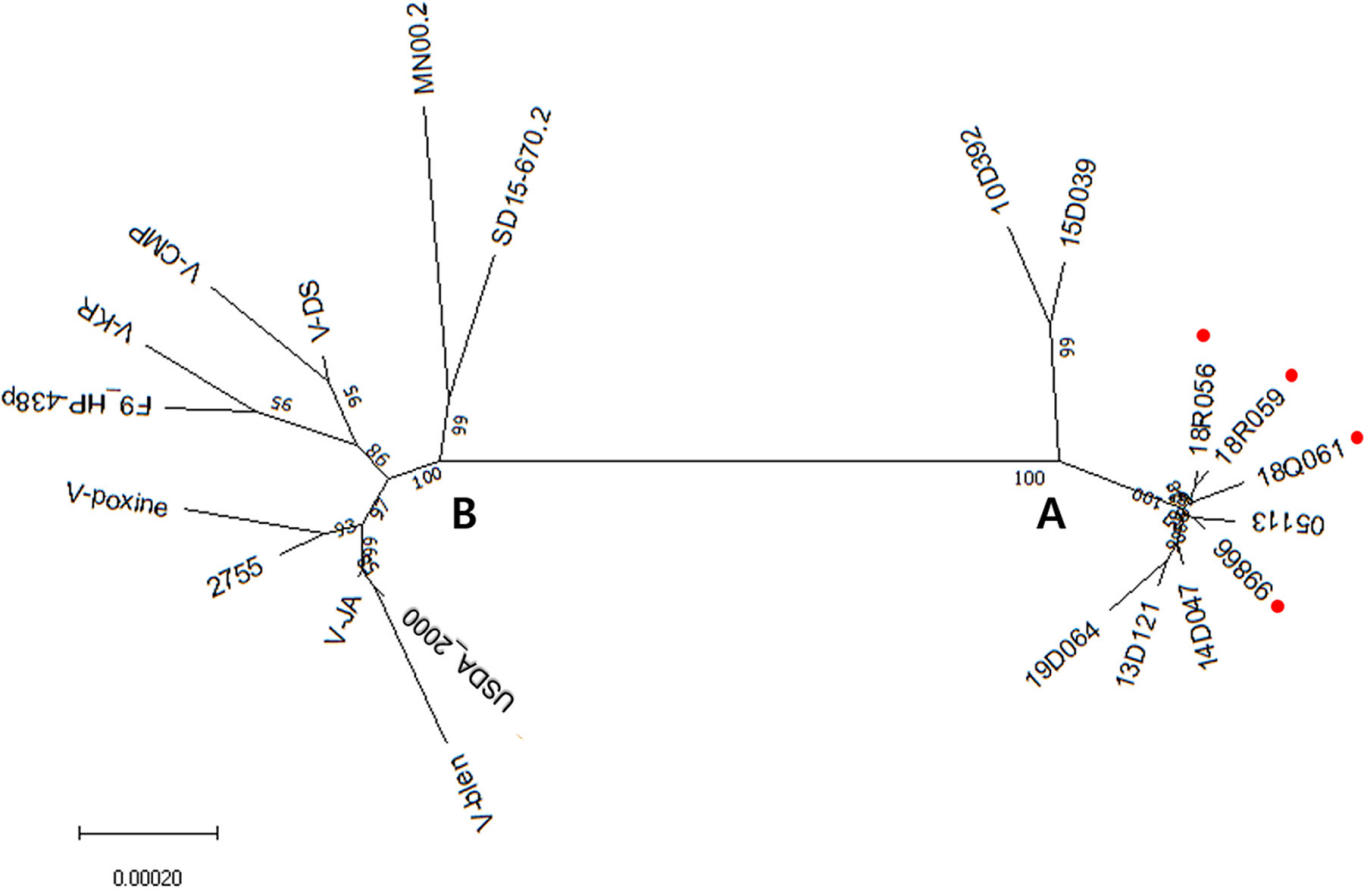
Phylogenetic tree of complete genomes of FPV using Neighbor-joining method. 21 FPVs are divided into two clades that represent clusters of isolates with origins in Korea (clade A) and North America-Europe (clade B). The Korean clade is divided into two subgroups with 10D392 and 15D39 strains being classified into a subgroup different from the other 8 strains. Strains isolated from chickens showing diphtheritic pox are indicated by a red circle.

The phylogenetic analysis of whole genomes divided the 21 FPVs into two clades that represented clusters of isolates with origins in Korea (clade A) and North America-Europe (clade B). Clade A was divided into two subgroups with the 10D392 and 15D39 strains classified into a subgroup different from the other eight strains. Four strains (99866, 18Q61, 18R056, and 18R059) isolated from chickens with diphtheritic pox were not divided into separate subgroups. Clade B was divided into three subgroups, with the MN00.2 and SD15-670.2 strains belonging to a subgroup different from the others. The remaining viruses were split into two subgroups: (i) V-CMP, V-DS, V-KR, and FP9 and (ii) V-JA, V-Poxine, V-Blen, USDA_2000, and 2755. V-CMP, V-DS, and V-KR strains were of the same origin as FP9. The V-POXINE strain was similar to the 2755 strain and the V-JA and V-BLEN strains were similar and appeared to be of the same origin as the USDA_2000 (Fig. 3). Phylogeny showed that FPVs have hardly evolved over time and there is no correlation between phenotype and phylogenetic association but appear to vary from region to region.

### Comparative genomics.

Forty putative proteins were selected (as described in Materials and Methods) for the comparative genomics analysis, of which 22 were functionally diverse: 11 of ankyrin repeat gene family proteins, 6 of Variola B22R, 3 of C-type lectin gene, and 2 of A-type inclusion proteins and the other 18 genes of each function. Twenty-one FPVs had at least one variation in one or the other ORFs, including gene truncation, gene loss, and/or different lengths of ORFs due to insertion/deletion/mutation of amino acids (aa) (Table 2 and S1).

**TABLE 2:** Summary of the comparative analysis of 40 putative proteins of FPV (see Table S1 for full details)

Genetic variation (amino acid mutation and/or insertion and/or deletion)	Gene	FPV ORF
Geographically distinct between Korean strain versus US strain	α-SNAP	11
	V-type Ig domain	17
	Ankyrin repeat gene family protein	18^*a*^, 115, 216, 223, 230, 242–243
	G protein-coupled receptor gene	27^*b*^
	Transforming growth factor-β	80^*a*^
	DNA polymerase	94^*c*^
	N1R/p28 gene family	124^*b*^
	Late transcription factor VLTF-4	142
	Molluscum contagious virus MC089L	145^*b*^
	Immunodominant virion protein	168^*b*^
	C-type lectin gene	239^*b*^
Common to four attenuated strains (V-CMP, V-DS, V-KR and FP9)	G protein-coupled receptor gene	27^*b*^
	Phosphopantothenate-cysteine	71
	DNA polymerase	94^*c*^
	Variola B22R	97
	N1R/p28 gene family	124^*b*^
	Molluscum contagious virus MC089L	145^*b*^
	Immunodominant virion protein	168^*b*^
	Early transcription factor, VETF-L	171
	A type inclusion protein	190, 191
	Ankyrin repeat gene family protein	219
	C-type lectin gene	239^*b*^
Unique in the diphtheritic type strains	Ankyrin repeat gene family protein	18^*a*^, 26
	Transforming growth factor-β	80^*a*^
	DNA polymerase	94^*c*^
	Variola B22R	98, 122

a ORF18 and 80 have genetic variations showing in the geographically distinct of FPVs and in only diphtheritic type strains.

b ORF 27, 124, 145, 168, and 239 have genetic differences showing in the geographically distinct and common to the attenuated strains.

c ORF 94 has genetic difference relating geographically distinct, showing in only diphtheritic type strains and common to the attenuated strains.

The genetic characteristics of viruses differed the most with their geographic variations. Ankyrin repeat gene family (11, 12) revealed that the Korean field strains differed from the U.S. strains in ORFs 18, 115, 216, 223, 230, 242, and 243. ORF 97, 98, 99, and 122, of the six ORFs belonging to the Variola B22R gene family, have multiple aa deletions or insertions or mutations in Korean strains, different from those in the U.S. strain, while the ORFs 107 and 123 have multiple genetic variations, without any specific pattern. We believe these genes are classified into the Variola B22R gene family based on their regional and host-specific differences, but not pathogenic ones. ORF 239, belonging to the C-type lectin gene, showed a clear difference (one aa mutation of K5R) between Korean and U.S. strains. The deletion of 117 to 163 aa of ORF 239 was observed in only four attenuated strains (V-CMP, V-DS, V-KR, and FP9). The genetic difference between attenuated strains and field strains was also found in 12 ORFs (ORFs 27, 71, 94, 97, 124, 145, 168, 171, 190, 191, 219, and 239), but these differences were characteristics that mainly appeared in the V-CMP, V-DS, V-KR, and FP9, and were not shown in other three vaccine strains (V-JA, V-POXINE, V-BLEN), presumed to be from the U.S. strains. Out of 12 ORFs, two genes (ORFs 124 and 168) were estimated to be associated with the virulence of FPV. ORF124 designated to N1R/p28 gene family was not encoded by four vaccine strains due to either truncation or deletion, and the other vaccine strains had an aa mutation or insertion, unlike field isolates. Immunodominant virion protein ORF 168, known to have variable size among the FPVs (13), was found to have a specific motif that consisted of 12 aa (TSSSVSGLAPQG). This motif showed 82% aa identity with that of the SDR family NAD(P)-dependent oxidoreductase. The FPVs analyzed in this study had a different number of motifs. Vaccines or attenuated strains have 6 or 7 motifs, and the USA_2000 strain has 4 motifs; strains 15D039, 18Q061, and 2755 have 1 motif, but nine Korean strains have no motifs. ORF 190 and 191, homologs of poxvirus A-type inclusion protein, are thought to be of significance for viral transmission in nature (14, 15). V-CMP, V-DS, V-KR, and FP9 strains had common ORFs 190 and 191 showing aa deletion and mutation in field strains, but both proteins of V-JA, V-POXINE, and V-BLEN strains were the same as those of the field isolates. ORF 71 was newly annotated as a phosphopantothenate-cysteine ligase (49.7% of aa identity to XP024062572) in this study. ORF 71 was a single protein of 289 aa in the most of FPVs but was truncated into 2 or 3 ORFs in the strains V-CMP, V-DS, V-KR, and FP9 (Table S1 and S2).

### Genetic differences of the attenuated viruses.

Three Korean veterinary drug manufacturers have been producing live FPV vaccines since the 1960s, but they have no information on the origin of the strain exclusive of the term “2755” in their archival documents. This study began when an archived virus labeled “strain 2755” was discovered in our laboratory’s −80°C freezer. Jeon et al. (9) reported that the 2755 strain was distributed by Cunningham at Michigan University, before 1963. Three manufacturers passaged and attenuated this strain, and authorized for use in 1969, 1976, and 1985, respectively.

Analysis of the complete genomes showed that three vaccine strains (V-CMP, V-DS, and V-KR) have low nucleotide (nt) identity (87.33 to 90.16%) compared to the 2755 strain assumed to be their origin (Fig. 1). Instead, they showed the same distinctive fingerprint of deletions (ORFs 97,124 to 125, 158 to 159, and 222) as the FP9 strain in genome alignment and higher nt identity (90.88 to 92.74%) with FP9 (Fig. 1 and 2). V-CMP, V-DS, V-KR, and FP9 strains had 259, 258, 260, and 253 ORFs, respectively, which were different from that of the 2755 strain (265 ORF). These attenuated strains have no *gag*, *pol*, and *env* genes of REV, but have truncated genes (Photolyase^158^, vaccinia A47L homolog^221^, Ankyrin repeat gene^222^, C-type lectin genes^239^), fragmented genes (T10 gene^70^, A-type inclusion protein^190^), synthesized genes (G protein-coupled receptor gene^27^, Serpin gene^40^), and gene losses (variola B22R gene^97^, two N1R/p28 genes^124 and 159^, V-type Ig domain^125^, ubiquitin-like gene^70^). Two deletions at 1633 bp and 1689 bp in noncoding regions at the 5′- and 3′-ends, respectively, and gene losses/additions of multiple hypothetical proteins are shown, compared with the 2755 strain. However, multiple deletions (C-type lectin genes^1 and 260^, Ankyrin repeat gene^241 to 247^) seen in FP9 strain were not seen in V-CMP, V-DS, V-KR, and 2755 strain (supplementary data T2).

### Genetic difference between diphtheritic and cutaneous fowlpox virus.

**FIG 4:**
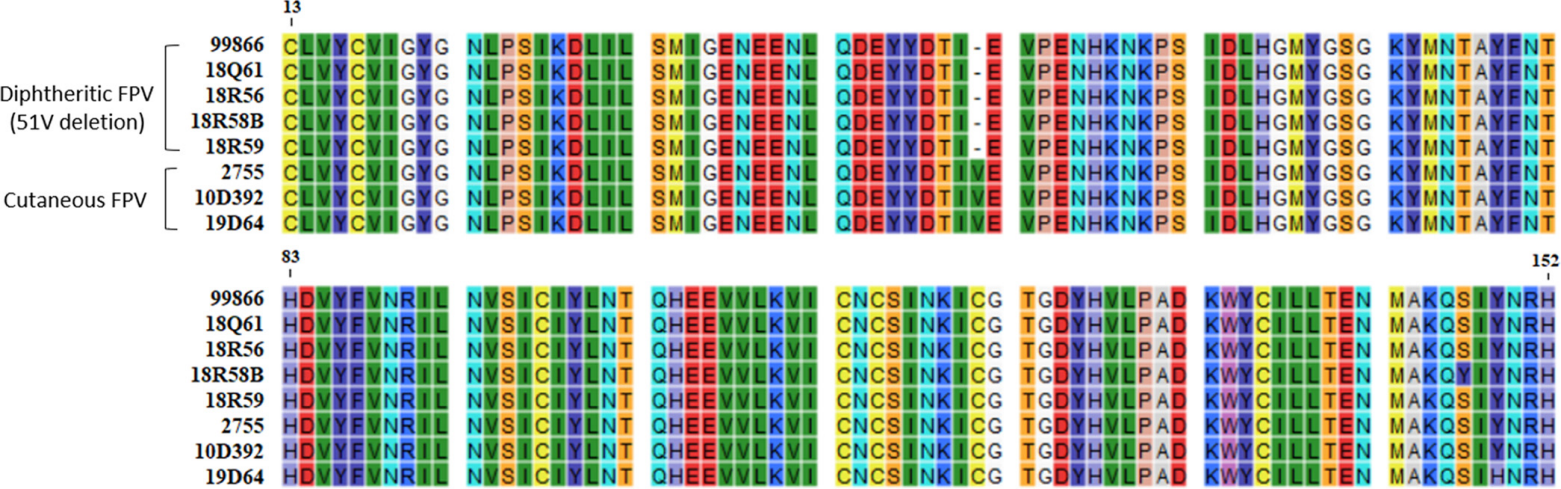
Alignment sequence of transforming growth factor-β (TGF-β) gene of the FPVs using Sanger sequencing.

Four strains (99866, 18Q061, 18R056, and 18R059) isolated from the trachea of chickens, having diphtheritic lesions, had a valine (V) deletion at aa position 51 of ORF 80 of the predicted transforming growth factor (TGF)-β gene. Sanger sequencing of partial TGF-β gene was performed to determine whether valine deletion is characteristic of diphtheritic pox virus. Diphtheritic FPVs, including another isolate that was excluded from the whole-genome analysis, showed the same results (Fig. 4). This result helps us identify specific genetic variations that would help differentiate the diphtheritic virus from the cutaneous virus, but it is not known whether the V deletion location is a functionally important region of homologous proteins. The 18Q061, 18R056 and 18R059 strains had point mutation in four genes: M252I in ORF 18, E333K in ORF 26, P721T in ORF 94, and R110Q in ORF 122. A mutation resulting in a substitution of tyrosine (Y) by cysteine (C) at the aa position 1414 of ORF 98 (Variola B22R) was found in the 18Q061 and 18R059 strains. Since aa variation in these five genes was caused by only a single base change, although these differences might also be involved in diphtheritic FPV, no further analysis was done (Table 2 and S1).

### Genetic characteristics of REV inserted into the genome of FPV field strains.

**FIG 5:**
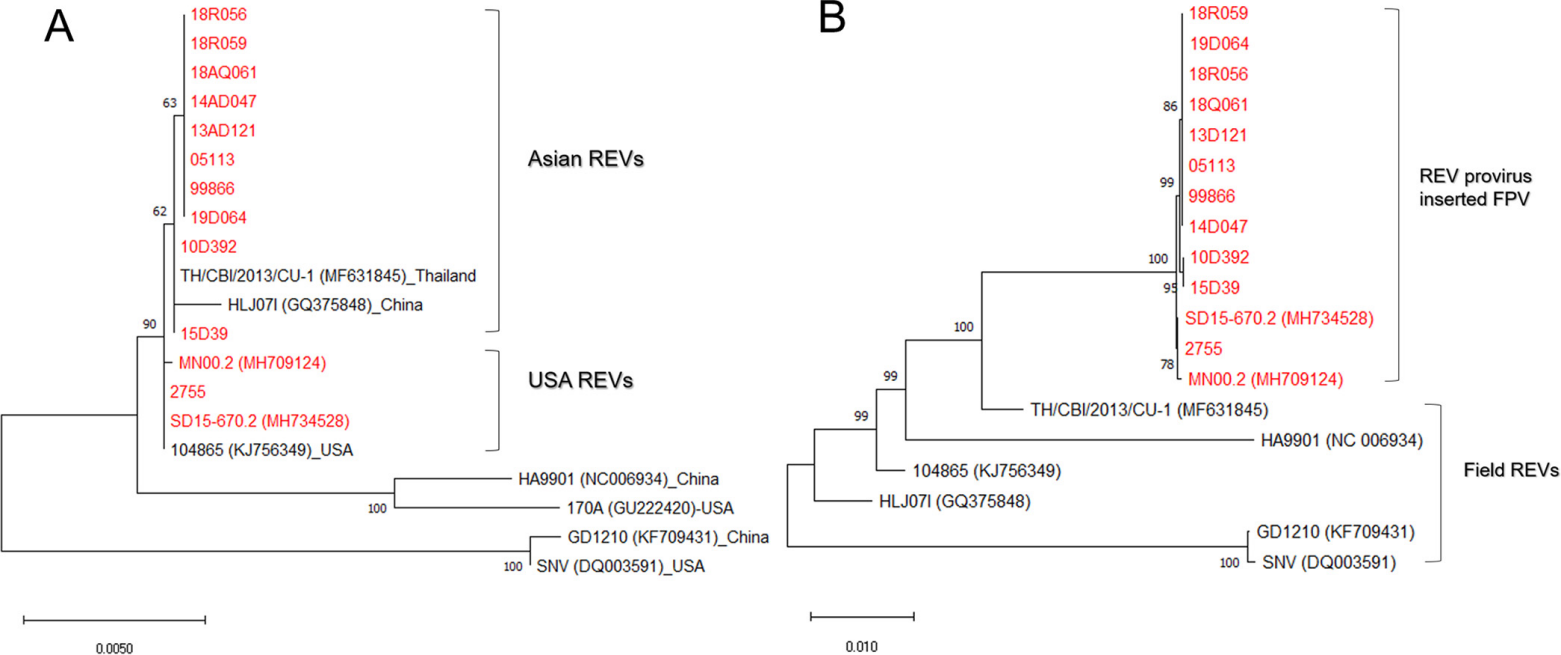
Phylogenetic tree of *env* genes (A) and complete genomes (B) of REV inserted in FPV virus using Neighbor-joining method. REV provirus inserted FPV, indicated by red, refers to nearly intact REV integrated in the genome of field FPV strains.

Thirteen field strains retained a full-length REV provirus, and seven vaccine strains contained portions of REV long terminal repeats. The complete genomes of REV inserted into FPV showed a high nt identity of more than 98.11%, and three U.S. strains had one aa point mutation in the gag and env proteins (Table 3). REVs inserted within the genomes of FPV were classified differently from field REVs in the phylogenetic tree of the complete genomes, but in the phylogeny of the *env* gene, REV strains within the genomes of FPV showed regional similarity to field REVs (Fig. 5).

**TABLE 3:** The different amino acids of REV inserted FPV tested in this study^*a*^

Strain	Gag protein 500 aa		Polymerase 1152 aa	Envelope glycoprotein 587 aa	
Position of aa	45	136	139	318	540
99866	F	M	V	T	F
05113	•	•	•	•	•
10D392	•	•	•	•	•
13D121	•	•	•	•	•
14D047	•	•	•	•	•
15D039	•	•	•	•	•
18Q061	•	•	•	•	•
18R056	•	•	•	•	•
18R059	•	•	•	•	•
19D064	•	•	•	•	•
2755	L	I	•	•	L
MN00.2	L	•	I	I	L
SD15-670.2	L	•	•	•	L

a F, Phe; I, Ile; L, Leu; M, Met; T, Thr; V, Val. • means that it is not different from the aminoacid in the above line.

### The pathogenicity of fowlpox virus.

**FIG 6:**
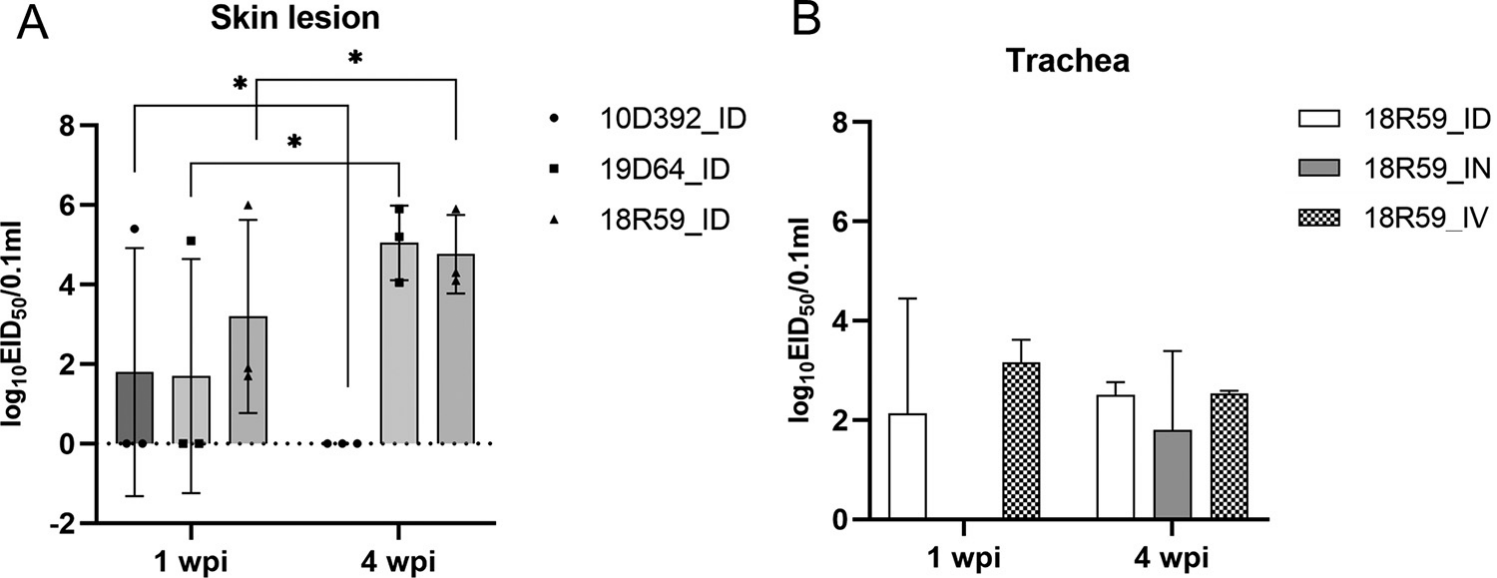
Fowlpox viral loads of skin lesions (A) of the chickens inoculated with 18R59, 10D392, and 19D64 strains (Experiment A) and viral loads of trachea (B) of chickens inoculated with 18R59 strain via different routes (Experiment B) using real-time PCR. The error bars represent the standard deviation of the mean. Viral loads were calculated by y = −3.5883× + 38.866, R2 = 0.9938 (The limit of detection < 1.3 log_10_EID_50_/0.1 mL).

To compare the pathogenicity between different strains, an *in vivo* pathogenicity trial was performed in SPF chickens without previous immunization (Experiment A). All chickens were intradermally inoculated with the three FPV strains (10D392, 19D64, and 18R59). The chickens showed cutaneous FP-like redness and crusting on the comb, the skin around the eyes, and beak at 1 to 4 weeks postinoculation (wpi). Symptoms characterized by strains 19D64 and 18R59 lasted up to 4 wpi, and high viral loads of skin lesions were maintained until 4 wpi. However, strain 10D392, classified into a subgroup different from the others in the genomic analysis, was detected at lower viral loads from inoculated chickens and the symptoms also lasted for a shorter duration. Diphtheritic lesions were observed only in chickens infected with the 18R59 strain (Table 4 and Fig. 6A). Experiment B was performed to find out whether diphtheritic lesions were caused by different infection routes or not. As a result of comparison between intradermal, intranasal, and intravenous inoculations with strain 18R59, tracheal lesions were observed in all chickens infected with strain 18R59 regardless of the route of infection. The clinical signs and death by the diphtheritic strain appeared at 2 to 3 wpi, and the viral loads in tracheal samples were identified at 1 wpi in the groups of intradermal and intravenous routes and remained low until 4 wpi. (Table 4 and Fig. 6B). Experiment C involved the evaluation of three commercial vaccines to find out if they have any efficacy against the recent field isolates. This was because FP has recently reemerged in vaccinated flocks, and a genetic difference is observed between the vaccine strains and the field strains in the genomic analysis. Each immunized group had no lesions or viral detection of FPV for 4 weeks postchallenge by field strains, regardless of the inoculation route and challenge strains (Table 4). This suggests that vaccine-induced antibodies are sufficient to prevent FP onset, although field viruses challenged are from a different genogroup than vaccine strains.

**TABLE 4:** Detection of FPV and REV and humoral antibody response to REV in the chickens, vaccinated or not after infection with field FPV strains

Test set^*a*^	Challenge strain	Route^*b*^	FPV detection in skin lesion		FPV detection in trachea		REV detection in thymus		Anti-REV antibody positive	
			1 wpi*^†^*	4 wpi	1 wpi	4 wpi	1 wpi	4 wpi	2 wpi	4 wpi
A	PBS	ID	0/3	0/3	0/3	0/3	0/3	0/3	0/9	0/6
A	10D392	ID	1/3	0/3	0/3	0/3	3/3	1/3	9/9	7/7
A, C	19D64	ID	1/3	3/3	0/3	0/3	0/3	1/3	7/9	9/9
A, B	18R59	ID	1/3	3/3	2/3	3/3	2/3	1/3	7/9	8/8
B	18R59	IV	2/3	2/3	3/3	3/3	3/3	0/3	9/9	3/3
B	18R59	IN	0/3	1/2	0/3	2/2	0/3	0/2	5/7	2/2
C	18R59 after V-kr vaccination	IN	0/3	0/3	nt^*c*^	0/3	1/3	nt	0/9	2/6
C	19D64 after V-cmp vaccination	ID	0/3	0/3	0/3	0/3	3/3	0/3	6/6	6/6
C	19D64 after V-blen vaccination	ID	0/3	0/3	0/3	0/3	2/3	0/3	6/6	1/6

a A test was performed to compare pathogenicity between different strains; B test to compare pathogenicity between different inoculation routes; C test to evaluate vaccine efficacy.

b ID, intradermal; IN, intranasal; IV, intravenous; WPI, weeks postinoculation.

c nt, not tested.

Meanwhile, the majority of virus-exposed chickens, vaccinated or not, produced anti-REV-specific antibodies from 2 wpi from the challenge with three field strains carrying near-full-length REV provirus. REV was detected at 1 wpi in thymus of the chickens infected with FPV and persisted up to 4 wpi in a few chickens (Table 4). This result indicated that the REV in the FPV field strains was replication competent, although no chicken had clinical signs of REV infection during the experiment.

## DISCUSSION

Despite advances in sequencing technology, studies on the genetic variation of FPV are very scarce owing to its large genome size, high homology, and less variation (2, 16, 17). This study was the first to analyze the complete genomes of 21 FPVs and showed that genetic differences in FPVs correlated with geographical distribution, attenuation of some vaccine strains, and the onset of diphtheritic lesions. However, viral evolution and mutations occurring with time were almost nonexistent in this analysis, even after decades of FP outbreaks.

Previous studies estimated that a few genes are associated with phenotypic differences in FPV (3) and the genetic divergence of different avipoxviruses in the terminal regions (18, 19). However, most of these genes are highly conserved between FPVs and comparison with other avipoxviruses with host-dependent specificity makes it difficult to predict gene function. Comparative analyses of various FPV strains and pathogenicity studies allowed us to elucidate the specific genes. The 12 genes (ORFs 27, 71, 94, 97, 124, 145, 168, 171, 190, 191, 219, 239) that showed genetic differences between attenuated strains and field strains were thought to influence viral pathogenicity regardless of viral replication, and 6 genes (ORFs 18, 26, 80, 94, 98, 122) showing genetic variation between diphtheritic FPV and cutaneous FPV were assumed to be relation to phenotypic differences.

Gene loss/addition events of poxviruses have been known to occur over the course of viral evolution (20, 21), because these differences may be related to pathogenicity, virulence, or replication of the virus. Until now, only the complete sequence of FP9 (HP-438p), the attenuated virus, was known that has multiple differences, including deletions, insertions, and mutations, compared to those of USDA_2000 and HP-1 (FP9 counterpart) (5). Unfortunately, there was no complete sequence of HP-1 and only a few genes of this virus were compared. In this study, we determined the full-length sequences of several commercial FPV vaccine strains and analyzed their phylogeny to reveal vaccine origins and elucidate genetic characteristics that are different from field isolates. Similar to FP9, the extensively passaged strains V-CMP, V-DS, and V-KR showed multiple mutations that might be characteristic of FPV evolution, related to viral replication. Although strain 2755 was the origin of V-CMP, V-DS, and V-KR strains, according to archival documents of veterinary pharmaceutical companies, FP9 cannot be completely ruled out as their origin.

A few vaccine strains had a difference of more than 10% of nt identity with the field virus and variable genetic differences in multiple genes, but protective effects against virulent field viruses were observed. Interestingly, strain V-JA was very similar to the USDA_2000 strain, except for the REV and N1R/p28 genes (ORF 124). The N1R/p28 gene, known to promote virulence by suppressing apoptosis (22), can be considered a key gene regulating viral pathogenicity. An intact REV provirus inserted into the FPV was observed in 1997 (23), but whether this was due to an iatrogenic contamination in the laboratory or natural events over evolutionary time has been controversial (2). Our analysis of the genomes of 13 field FPVs, including the 1960s virus, showed that the integrated REV provirus appeared to be an ancestral insertion and seemed to be dormant in the FPV, without genetic mutation, for a long time.

In this study, the majority of chickens infected by three field isolates developed high levels of antibodies against REV and were positive for REV antigen, with or without vaccination. The findings agree with previous studies on Australian field FPVs and FPVs with intact REV (23, 24). The genomes of the field FPVs causing outbreaks of FP in vaccinated flocks mostly contain an integrated, nearly intact provirus of REV, which has played a role in the development of immunosuppression in young chickens (8, 24). This retention of REV may later be involved in a reemerging FP in flocks with insufficient immunity, but it was difficult to confirm immunosuppression by REV inserted into field FPV or the enhancement of pathogenicity of FPV in the short period experiment of this study.

Our study suggests key genomic features that differentiate diphtheritic poxviruses from the relatively less pathogenic cutaneous viruses. In particular, valine (V) deletion, commonly identified in the TGF-β genes of five diphtheritic FPVs, was expected to result from multiple deletion events. A homolog of eukaryotic TGF-β, encoded ORF 80, is known to stimulate connective tissue growth and differentiation within the FPV genome and to promote rhinovirus replication in bronchial epithelial cells by suppressing the innate immune response (3, 8, 25). Further studies to determine their loss or gain of function as a pathogenic mechanism in the expression of diphtheritic lesions are needed.

## MATERIALS AND METHODS

### Viruses.

Cutaneous pox and diphtheritic fowlpox were diagnosed based on microscopic lesions in the skin scab and trachea, characterized by marked proliferation of the epithelium that contained eosinophilic cytoplasmic inclusion bodies. 10 FPVs were selected from cases diagnosed with FP in the Republic of Korea between 1999 and 2019. Eleven field strains, including the 2755 strain provided by the University of Michigan in the 1960s (9), were propagated using the chorioallantoic membrane (CAM) of specific pathogen-free embryonated eggs. Five days after egg inoculation, the collected CAM was processed promptly via blending into a 10% homogenate in sterile phosphate-buffered saline (PBS) containing 0.4 mg gentamicin per mL and centrifuged at 3,500 rpm and 13,000 rpm for 10 min each. To remove large particles and bacteria, the supernatants were filtered with 0.45 and 0.22 μm syringe filters and stored at −80°C until analysis. Six vaccines distributed in Korea were examined to obtain the whole-genome sequence and analyzed to elucidate the genetic diversity of the complete genome.

### Genome sequencing, assembly, and annotation.

Total DNA was extracted from purified samples using the DNeasy blood and tissue kit (Qiagen, Hilden, Germany). Library preparation and barcoding were performed using the Nextera DNA Flex Library Prep kit and Nextera DNA CD Indexes (Illumina, CA, USA), according to the manufacturer’s instructions. An Illumina MiSeq instrument (Illumina) was used for whole-genome sequencing. After base calling, paired-end reads were exported as FASTQ files for assembly. A MinION Flow Cell (R9.4.1; Oxford Nanopore Technology) was used to obtain the long reads of five strains (V-POXINE, V-BLEN, V-DS, V-KR, and V-JA). MinION library preparation was performed using the 1D^2^ library preparation kit (SQK-LSK308; Oxford Nanopore Technology), according to the manufacturer’s instructions. The distribution of size and concentration of genomic DNA and library was checked on a TapeStation 4150 instrument (Agilent Technologies, Santa Clara, CA), using high-sensitivity D1000 reagents and ScreenTapes (Agilent Technologies), and on a Qubit fluorometer using the double-stranded DNA HS assay kit (Thermo Fisher Scientific Waltham, MA), respectively. CLC Genomics Workbench 20.0.2 (CLC Bio, Aarhus, Denmark) was used for adapter trimming, host genome mapping, *de novo* assembly, and reference mapping. The trimmed reads were assembled after the host genome (galGal4) was filtered and aligned. *De novo* assembly of short reads was performed. Because FPVs have identical ITR regions, the 5′-end (nt 1 to 20,000) and 3′-end (nt 251,057 to 288,539) were assembled to reference sequences (GenBank accession number AF198100.1). The longest contigs and the 5′ and 3′ sequences assembled by the reference genome sequence were combined to make the whole genome. However, for the five strains, *de novo* assembly using long reads and short-read polishing was performed (Table S3). We obtained a total of 17 FPV genome sequences that were then annotated using four reference strains; USDA_2000 (AF198100), MN00.2 (MH709124), SD15-670.2 (MH734528), and FP9-passage 438 (AJ581527.1), and Prokka v1.13 (10). To define the function of each gene, we performed local BLAST+ using the previously annotated FP virus genome (NC_024447.1) as a database with an e-value cutoff 10^−6^. If there were no search results, the GenBank nr DB was used.

### Comparative genomics analysis.

PGAP in the Pan-Genome Analysis Web Server (http://pgaweb.vlcc.cn/) was used for pan-genome analysis, homologous gene clustering (ortholog clustering), homologous cluster variation analysis, and evolution analysis (26). Prokka output files (.gbk, .ffn, and faa) were converted into the appropriate input file formats (.function, .nuc, and .pep) using Python 3. MultiParanoid method was used to find orthologous genes in multiple proteoms. To compare the identities of each homologous gene, the gene sequences were extracted using Cluster ID in the PGAP output. The MAFFT program was used to generate large-scale multiple alignments of the DNA sequences (27). Phylogenetic trees were generated by the neighbor-joining method using MEGA X software with 1000 bootstrap replications (28). The sequence lists were pairwise compared to calculated percent identity against consensus sequence using CLC Genomic Workbench 20.0.2 (CLC Bio, Aarhus, Denmark). Through the analysis of more than 260 clusters for each of the 21 viruses, 35 clusters, excluding hypothetical proteins, were selected from among 50 clusters with indel bases exceeding zero. In addition, we added five clusters with more than four nonsynonymous mutations among those with an indel base of zero because nonsynonymous mutations can change the aa. Synonymous mutations were not considered because they were minor and did not change anything (29). The sequences of 40 ORFs were selected, extracted using Python, and aligned using CLC Main Workbench 7.7.2 (CLC Bio, Aarhus, Denmark) to perform comparative genomics. To avoid confusion, the number of ORFs used in this study followed the ORFs designated by Afonso et al. (3).

### Direct sequencing of the partial TGF- β gene.

PCR of the partial TGF-β gene was performed using the primer pair ORF 80F (5′-TCTCACCGGTCCTATGAGGTT-3′) and ORF 80R (5′-ATGCGTCAAGCGTTAAAATGGT-3′). PCR was performed using Black PCR Premix QM (2×) (Cosmogenetech, South Korea) under the following PCR conditions: 94°C for 3 min; 35 cycles at 94°C for 1 min, 57°C for 1 min, 72°C for 1 min, and 72°C for 5 min. Direct sequencing of the PCR product was performed at Macrogen (Seoul, South Korea) using an ABI 3730 XL DNA sequencer (Applied Biosystems, Foster City, CA, USA). The sequences of the isolated viruses were aligned and edited with CLC Main Workbench 7.7.2 (CLC Bio, Aarhus, Denmark).

### Experimental infection in chickens.

The Animal Ethics Committee of the Animal and Plant Quarantine Agency approved this study (Animal Ethics Approval: 2020-482). A total of 108 7-week-old SPF white leghorn broiler chickens (*Gallus domesticus*) were caged into nine groups (Table 3). Three field strains (10D392, 19D64, and 18R59) were propagated and titrated onto the chorioallantoic membranes of embryonated eggs. A dose of 0.2 mL (5.5 logEID_50_/0.1 mL) of each strain was inoculated intradermally, intranasally, and intravenously into the 12 chickens in every group. Three commercial vaccines, Himmvac FP vaccine (KBNP, South Korea), Pro-vac FP vaccine (Komipharm, South Korea), and Pox-Blen FP vaccine (Boehringer Ingelheim, USA), were administered to 5-week-old chickens by the wing-web method with a needle applicator carrying approximately 15 μL of vaccine. At 14 days postvaccination, all vaccinated chickens were challenged with strains 19D64 or 18R59. All groups were maintained in negative pressure isolators with a filtered air ventilation system and observed daily for clinical signs and mortality. At 7-, 14-, 21-, and 28-days postchallenge, three randomly chosen chickens were euthanized from each group for necropsy examination, and the trachea and skin were collected for viral detection using separate scissors to prevent cross-contamination. DNA was extracted from tissues using a commercial QIAamp DNeasy blood and tissue kit (Qiagen, Germany), and viral loads were determined using qPCR (30, 31). All statistical analyses were performed using GraphPad Prism version 9.2.0 (GraphPad Software Inc., Sand Diego, CA, USA). Two-tailed Student’s *t-*tests were performed to compare viral loads. Differences were considered significant at *P < *0.0001(*). Blood samples were obtained from the jugular vein of all chickens at 7-day intervals after inoculation, and all sera were diluted 500-fold using a commercial REV ELISA kit (IDEXX, USA), according to the manufacturer’s instructions. Serum samples with S/P ratios ≤ 0.50 were considered negative, while S/P ratios > 0.50 were considered positive.

### Data and availability.

All data, code, and materials used in the analysis must be available in some form to any researcher.
